# Treatment of convulsive status epilepticus in Brazil: a review

**DOI:** 10.1055/s-0045-1801872

**Published:** 2025-02-11

**Authors:** Luis Otavio Caboclo

**Affiliations:** 1 Hospital Israelita Albert Einstein, São Paulo SP, Brazil.

**Keywords:** Status Epilepticus, Refractory, Super-Refractory, Antiseizure Medications

## Abstract

Status epilepticus (SE) is the most severe presentation of epilepsy. Currently, SE is defined according to 2 sequential time frames: time 1, after which it is unlikely that the seizure will resolve spontaneously, therefore requiring the initiation of therapy; and time 2, when long-term consequences become more likely. For convulsive SE, these time frames are well defined: 5 minutes for time 1 and 30 minutes for time 2. “Time is brain” in the treatment of SE, as delays in diagnosis and treatment are associated with worse outcomes. After clinical stabilization, the first step is the administration of intravenous (IV) benzodiazepines. Rapid initiation of treatment and use of appropriate dosing are more important than the selection of a specific benzodiazepine. Following this, treatment continues with the use of an IV antiseizure medication (ASM). In Brazil, the recommended options available are phenytoin and levetiracetam. Status epilepticus is considered refractory to treatment if seizures persist after the administration of benzodiazepines and IV ASM. The cornerstone of this stage is the induction of therapeutic coma using IV anesthetic drugs (IVADs), although evidence is limited regarding the choice among midazolam, propofol, or barbiturates. Super-refractory SE is defined when seizures persist despite continuous infusion of IVADs or recur after these drugs are tapered. There is very limited data regarding the treatment of super-refractory SE. In the absence of randomized controlled trials, treatment should be guided by the physician's experience, clinical judgment, and established therapeutic options from previous reports.

## INTRODUCTION

Status epilepticus (SE) is considered the most extreme form of seizure and the most severe presentation of epilepsy. It consists in abnormally-prolonged or recurrent seizures without recovery of the baseline status between them. Previous classifications of seizures defined SE as a seizure that “persists for a sufficient length of time or is repeated frequently enough that recovery between attacks does not occur.” 1 More recently, SE has been defined considering two distinct time frames, which will be discussed in the following sections.

Status epilepticus affects all age groups, though with different etiologies and outcomes. It causes higher mortality and morbidity in elderly people, while it has better outcomes in children and adolescents.

## DEFINITION

Status epilepticus has long been defined as abnormally-prolonged or recurrent seizures. 2 In 2015, the International League Against Epilepsy (ILAE) Task Force on Classification of Status Epilepticus published a definition and classification of SE. 3 According to the current ILAE proposal, SE is defined as “a condition resulting either from the failure of the mechanisms responsible for seizure termination or from the initiation of mechanisms which lead to abnormally-prolonged seizures (after time point t1). It is a condition which can have long-term consequences (after time point t2), including neuronal death, neuronal injury, and alteration of neuronal networks, depending on the type and duration of seizures”. Therefore, two time points are defined in the evolution of prolonged seizures. For the most severe type of SE – convulsive SE –, these time points are best defined, and have clinical significance: time 1 (t1) is 5 minutes, as after this time it is unlikely that the seizure will resolve spontaneously, therefore requiring initiation of therapy; and time 2 (t2) is 30 minutes, as seizures that last beyond this time are more likely to cause long-term consequences. From a clinical point of view, one should start treatment at t1, aggressively enough so that the seizure does not last until t2. For other types of seizures, evidence supporting these time frames is less conclusive ( Table 1 ).

**Table 1:** Time points t1 and t2 according to seizure type in status epilepticus (SE)

	t1, when a seizure is likely to be prolonged	t2, when a seizure may cause long-term consequences
Convulsive SE	5 minutes	30 minutes
Focal SE	10 minutes	> 60 minutes
Absent SE	10–15 minutes	Unknown

Note: Adapted from Trinka et al. 3

The report of the ILAE Task Force 3 also proposes 4 axes for classification of SE: semiology; etiology; electroencephalogram (EEG) correlates; and age.

With respect to semiology, two criteria are used to define the type of SE: the presence or absence of prominent motor symptoms and the degree of consciousness impairment. In *convulsive SE* , there are prominent motor symptoms along with impairment of consciousness, whereas in *nonconvulsive SE* consciousness impairment occurs without accompanying motor symptoms. Most of the data on the treatment of SE is based on studies addressing convulsive SE; the evidence for the treatment of nonconvulsive SE is much scarcer. The underlying cause of SE is categorized into known etiologies, when SE is caused by a known disorder, or unknown, or cryptogenic, if the etiology is not defined after the diagnostic investigation. Etiologies known to cause SE include acute causes, such as stroke, encephalitis, intoxication, and others; remote causes, including poststroke and posttraumatic; and progressive diseases, such as brain tumors and neurodegenerative diseases. Status epilepticus can also occur in the context of defined electroclinical syndromes, in which SE is a severe presentation of seizures.

## EPIDEMIOLOGY

The annual incidence of SE ranges from 14 to 35 per 100 thousand children 4 5 and from 5 to 36 per 100 thousand adults, 6 although different methodologies in various studies, as well as the introduction of the new diagnostic criteria and classification, 7 limit the comparison of results among the epidemiological studies. Most epidemiological studies refer to convulsive SE, so data reflects more likely this type of SE. The incidence of SE, as that of epilepsy, presents a bimodal age distribution: 7 8 it is higher in children and in older adults, with different causes in the two groups; in children, febrile illnesses constitute the most common cause of SE, while in adults, stroke is the leading cause. 6 8 9

## TREATMENT

Status epilepticus is a common neurological emergency, with high morbidity and mortality, especially in its convulsive form. Early recognition of this condition should lead to prompt initiation of therapy, as early treatment is associated with improved outcomes. 10

Most data on the treatment of SE pertains to convulsive SE, as evidence on the treatment of nonconvulsive SE is much scarcer. In the following section, we will discuss the approach to children and adults with convulsive SE. The recommendations for treatment are largely based on previously published guidelines, 11 12 which have adapted to the current availability of antiseizure medications (ASMs) in Brazil.

## Initial approach

As with any medical emergency, the treatment of convulsive SE begins with an accurate diagnosis, followed by clinical stabilization and the administration of ASMs. The primary treatment objectives can be summarized in three steps, which should be performed simultaneously:

- Assessing if airway, breathing, and circulation are secure and adequate;
- Using ASMs to stop the seizure; and
- Identifying the etiology of seizures/SE and initiating specific treatment when possible.

Heart rate, blood pressure, breathing and pulse oximetry should be monitored in all patients, particularly in those with ongoing convulsions. Transient apnea and hypoxemia are common in patients with convulsive SE. In those with cyanosis and/or low oxygen levels by pulse oximetry, 100% oxygen should be provided, either by nasal catheter or bag-mask ventilation. If these measures fail to maintain adequate breathing and oxygenation, endotracheal intubation and mechanical ventilation should be considered. For intubation, an agent with antiseizure properties, such as propofol or midazolam, may be used.

To obtain blood samples for laboratory tests and to facilitate the intravenous (IV) administration of ASMs, peripheral IV access is required, preferably with at least two catheters. A finger-stick glucose test should be performed immediately, and if hypoglycemia is detected, it should be treated promptly with thiamine and a dextrose solution. Initial blood and urine tests are shown in Table 2 .

**Table 2:** Initial blood and urine tests

Complete blood count; Serum glucose; Serum electrolytes; Liver function tests; Urine and blood toxicology; Pregnancy test (blood or urine) in women of childbearing age; and Serum levels of antiseizure medications (in patients taking them)

A 12-lead electrocardiogram (ECG) should be performed as soon as possible, and cardiac troponin levels may be considered, as cardiac injury can complicate prolonged seizures and SE. 13

After clinical stabilization and seizure control with ASMs, a neuroimaging exam should be performed. Magnetic resonance imaging (MRI) of the brain is preferred over a head computed tomography (CT) to identify the cause of SE; however, CT is typically more accessible in the emergency department. If a brain imaging study excludes a brain lesion, a lumbar puncture for cerebrospinal fluid analysis may be performed if a central nervous system (CNS) infection is clinically suspected. If prompt brain imaging is not possible and there is a high suspicion of infection, empirical antibiotics should be initiated.

## Treatment with ASMs

Along with clinical stabilization of the patient, treatment of prolonged seizures and SE should be started immediately. The primary goal of treatment is to stop the seizure and prevent secondary neurological damage.

### *First-line treatment*

It has long been established that the first phase of treatment of convulsive SE is the rapid administration of benzodiazepines (BZDs). The seminal study by Treiman et al. 14 laid the foundations for the use of BZDs. In this multicenter study, adult patients with overt SE, defined as continuous generalized tonic-clonic (GTC) seizure lasting 10 minutes or longer, or 2 or more GTC seizures without full recovery of consciousness, were randomly allocated to 1 of 4 arms of treatment, all with IV medications: lorazepam (0.1 mg/kg); diazepam (0.15 mg/kg) followed by phenytoin (18 mg/kg); phenobarbital (18 mg/kg); and phenytoin alone (18 mg/kg). Lorazepam was found to be superior to phenytoin in stopping seizures within 20 minutes of IV infusion, with no recurrence prior to 60 minutes ( *p* = 0.001). Interestingly, lorazepam was no more efficacious than phenobarbital or diazepam and phenytoin, but the authors 14 argued it was easier to use. Since then, lorazepam remains as the first line of treatment in convulsive SE.

Which is the ideal BZD for the treatment of convulsive SE? The ideal agent should be safe, easy to administer, have long-lasting antiseizure effect, and no relevant side effects. 15 No single drug fulfills all of these requirements, and the choice of BZD must be based on existing guidelines and availability.

Intravenous lorazepam has been compared with IV diazepam in a class-II study, 16 and there was no significant difference with respect to seizure control. The Paramedic Treatment of Prolonged Seizures by Intramuscular Versus Intravenous Anticonvulsant Medications (RAMPART) trial, 17 published in 2012, brought a highly-important contribution to the issue of BZD choice in convulsive SE. In this study, intramuscular midazolam was compared with IV lorazepam in adults and children with SE, defined as convulsive seizures lasting more than 5 minutes and still occurring after paramedic arrival. Patients received either 10 mg (or 5 mg in children weighing between 13 and 40 kg) of intramuscular (IM) midazolam or 4 mg (or 2 mg in children weighing between 13 and 40 kg) of IV lorazepam. The aim of the trial was to demonstrate the noninferiority of IM midazolam when compared with IV lorazepam. Seizure cessation was achieved in 73% of subjects in the IM midazolam group, compared with 63% in the IV lorazepam group. 17 This result met the prespecified noninferiority requirement; moreover, it also demonstrated superiority of midazolam in patients without established IV access.

**Figure 1:**
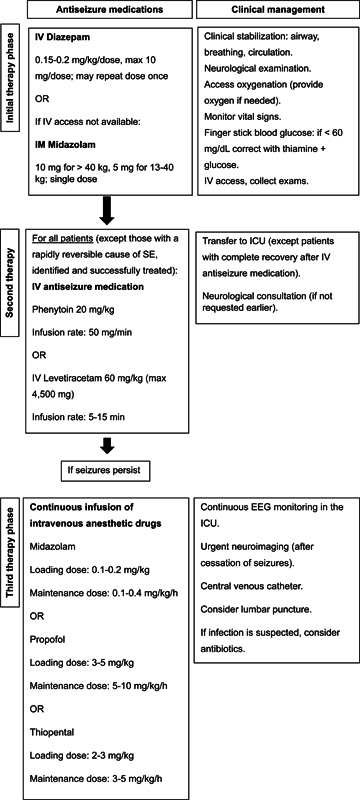
Treatment algorithm for convulsive status epilepticus.

Intravenous lorazepam is not available in Brazil. Therefore, IV diazepam or IM midazolam are recommended ( Figure 1 ). For patients without established IV access, IM midazolam is preferred to avoid delays in initiating the treatment. When IV access is not promptly available, midazolam may also be administered by nasal spray, which is has been approved by the United States Food and Drug Administration (FDA) for the acute treatment of seizure clusters in patients aged ≥ 12 years, or by oromucosal solution, which has been approved in Europe for prolonged seizures in children aged ≥ 3 months. 15 A recent systematic review 18 analyzed 12 studies with 929 patients who received intranasal midazolam for the treatment of prolonged seizures or SE, in single doses varying from 2.5 to 20 mg. Seizure termination was achieved in 72.7% of the patients, although seizures recurred in 36.5%. 18 Diazepam nasal spray has also been approved by the FDA for the acute treatment of seizure clusters in patients aged ≥ 6 years. 15 However, nasal midazolam and diazepam sprays, as well as midazolam oromucosal solutions, are not commercially available in Brazil.

As with stroke treatment, the aphorism “time is brain” should be kept in mind when treating SE, as treatment delays are independently associated with prolonged time until SE cessation. 10 Also, the dosing of the chosen BZD should be appropriate for each patient. In the clinical practice, treatment guidelines are not followed in a substantial proportion of patients. In a registry study of 1,049 patients, 10 the median latency between SE onset and the first treatment was of 30 minutes in patients with convulsive SE, and, in 76% of these cases, the bolus doses in the initial treatment step were lower than recommended. Treatment delays and underdosing were associated with failure to achieve SE cessation. 10 The Established Status Epilepticus Treatment Trial (ESETT) 19 studied the patterns of BZD use as the first-line treatment of SE and the association of BZD doses with response to second-line agents. In 460 patients who received 1,170 doses of BZDs (669 of lorazepam, 398 of midazolam, and 103 of diazepam), the first dose of the first BZD was lower than that recommended in the guideline in 76% of midazolam administrations and in 81% of lorazepam administrations. Among all administrations, > 85% of those of midazolam and > 76% of those of lorazepam were lower than recommended, and the authors 20 concluded that underdosing of BZD in SE treatment is both pervasive and difficult to remediate.

Given the current recommendations for BZD use as the first-line treatment of SE, it is worth emphasizing that appropriate timing and dosing are more important than the choice of the BZD itself, as earlier and more effective treatment of SE will lead to a better outcome.

### *Second-line treatment*

The second stage of treatment of SE begins when seizures persist after one or two doses of BZD. If the seizures respond to BZD, second-line medications should also be administered to prevent seizure recurrence, 11 as treatment with BZD alone carries a high risk of recurrence, 14 17 maybe with the exception of patients in whom a rapidly-reversible cause of SE has been identified and treated, such as severe hyponatremia of hypoglycemia; 11 with respect to hyponatremia, it is worth noting that the rapid treatment of this disturbance carries risk of complications, mainly osmotic demyelination syndrome.

In this stage, the mainstay of treatment is the use of IV ASMs. Currently, five ASMs can be loaded intravenously: phenytoin (or fosphenytoin), valproic acid, levetiracetam, phenobarbital, and lacosamide.

Until recently, the choice among these ASMs was guided by low-quality evidence, consisting in observational studies and small clinical trials, insufficient to recommend the choice of one specific ASM over the others. 21 22 In 2019, the ESETT 19 provided high-quality evidence for the choice of second-line treatment of SE. In this trial, 384 pediatric and adult patients with convulsive SE refractory to BZD were enrolled and randomly allocated to receive either fosphenytoin (20 mg/kg, phenytoin equivalents), valproate (40 mg/kg), or levetiracetam (60 mg/kg, all loaded intravenously). There was capping of weight-based dosing at 75 kg; therefore, heavier patients received a lower dose. The outcome of the study was cessation of SE and improvement in the level of consciousness at 60 minutes, which was achieved in 47% of the levetiracetam group, in 46% of the valproate group, and in 45% of the fosphenytoin group. In conclusion, the three ASMs were equally effective and yielded similar rates of adverse effects.

In the same year, two studies compared the effectiveness of phenytoin and levetiracetam in the treatment of convulsive SE refractory to BZD in children. The Levetiracetam versus Phenytoin for Second-Line Treatment of Convulsive Status Epilepticus in Children (ConSEPT) trial 23 was an open, multicenter, randomized trial conducted in centers in Australia and New Zealand. The Levetiracetam versus Phenytoin for Second-Line Treatment of Paediatric Convulsive Status Epilepticus (EcLiPsE) trial, 24 also an open, multicenter, randomized trial, was conducted in 30 centers in the United Kingdom. In both trials, children aged between 3 months and 16 years (ConSEPT) and between 6 months and 18 years (EcLiPsE) were randomly allocated to receive levetiracetam (40 mg/kg) or phenytoin (20 mg/kg). In ConSEPT, the primary outcome was clinical cessation of seizure activity 5 minutes after the completion of infusion in the study group, which was achieved in 68 (60%) patients in the phenytoin group and in 60 (50%) patients in the levetiracetam group ( *p* = 0.16, not statistically significant). In EcLiPsE, the primary outcome was time until cessation of convulsive SE. Convulsive SE was terminated in 106 (70%) children in the levetiracetam group and in 86 (64%) in the phenytoin group. The median time from randomization to cessation of convulsive SE was of 35 minutes in the levetiracetam group and 45 minutes in the phenytoin group ( *p* = 0.20, not statistically significant). In the two trials, 23 24 levetiracetam was equivalent to phenytoin with respect to seizure cessation and occurrence of adverse events. Given its safety profile and comparative ease of administration, levetiracetam could be an appropriate alternative to phenytoin as the first choice of second-line ASM in the treatment of pediatric convulsive SE.

Intravenous valproate is not available in Brazil. Therefore, based on the best available evidence, IV levetiracetam or IV phenytoin are recommended as second-line ASMs in convulsive SE. Figure 1 shows the suggested dosing for these medications.

Intravenous levetiracetam has recently become available for use in Brazil. This should lead to revisions in the current guidelines for the treatment of SE in the country. Levetiracetam is safe and easy to use. The loading dose may me infused in 5 to 15 minutes. 25 26 The rapid infusion (over 5 minutes) of doses up to 4,500 mg IV is safe and well tolerated. 25 27 Due to its safety profile and pharmacological characteristics, levetiracetam may be preferred in patients with liver failure, cardiac arrhythmias (which can be aggravated by IV phenytoin) or hemodynamic instability, although there is insufficient high-quality data to make these indications. The absence of significant drug interactions and a favorable tolerability profile are additional advantages when transitioning to long-term maintenance therapy. Table 3 details the doses of IV levetiracetam in children and adult patients, based on the aforementioned studies comparing levetiracetam to other ASMs. 19 23 24

**Table 3:** Levetiracetam dosing in children and adults with convulsive status epilepticus

Trial	Loading dose	Maximum dose	Age	Route	Infusion rate	Efficacy and safety
ESETT 19	60 mg/kg	4,500 mg	1–94 years	IV	10 minutes	Comparable to f-PHT and VPA
ConSEPT 23	40 mg/kg	3,000 mg	3 months–16 years	IV or IO	5 minutes	Comparable to PHT
EcLiPsE 24	40 mg/kg	3,000 mg	6 months–18 years	IV or IO	5 minutes	Comparable to PHT

**Abbreviations:** ConSEPT, Levetiracetam versus Phenytoin for Second-Line Treatment of Convulsive Status Epilepticus in Children; EcLiPsE Levetiracetam versus Phenytoin for Second-Line Treatment of Convulsive Status Epilepticus; ESETT Established Status Epilepticus Treatment Trial; f-PHT, fosphenytoin; IO, intraosseous; IV, intravenous; PHT, phenytoin; VPA, valproic acid.

There is less evidence regarding the use of phenobarbital and lacosamide as second-line treatment in convulsive SE. 21 22 Phenobarbital is very effective in the treatment of prolonged seizures and SE, with rates of seizure control of up to 60%. 21 22 28 29 However, phenobarbital causes prolonged sedation and carries a higher risk of hypotension and hypoventilation; therefore, it is less frequently used in the second stage of convulsive SE treatment. The initial dose is of 20 mg/kg, infused at a rate of 30 to 50 mg/minute. Ιntubation may be required to protect the airway, especially if phenobarbital is used after BZD. Intravenous lacosamide (200–400 mg IV bolus) has been used as an alternative for the second-line treatment of convulsive SE. 30 31 32 It is usually well tolerated, although atrioventricular block has rarely been reported; 30 ECG monitoring is recommended, especially when it is used along with other drugs that may prolong the PR interval. A systematic review 33 has suggested that lacosamide has an overall efficacy rate of 57% in treating SE and is more effective in focal motor SE. Lacosamide is comparable to fosphenytoin for the treatment of refractory nonconvulsive ѕeizures, 34 but evidence supporting its use in convulsive SE is lacking. 21 29 As of 2024, lacosamide has not been approved for the treatment of SE in Brazil.

## Refractory status epilepticus

Refractory SE is defined as seizures persisting despite the administration of at least two appropriately-selected and dosed parenteral medications, including a BZD. 11 35 No specific seizure duration is required. Refractory SE occurs in 1/5 to 1/4 cases of SE, and it causes elevated morbidity and mortality. 36 37 38 39 40

For most cases of refractory convulsive SE, continuous infusion of IV anesthetic drugs (IVADs) should be initiated. The patient should be transferred to the intensive care unit (ICU), as endotracheal intubation and mechanical ventilation are likely to be needed. A neurological consultation should be requested, and, if possible, continuous EEG monitoring should be initiated, particularly in patients sedated with continuous IVADs.

The IVADs used for SE treatment are midazolam, propofol, and pentobarbital/thiopental. In Brazil, thiopental is the barbiturate available for continuous infusion. In some cases, ketamine may also be indicated. Although there is insufficient data to support the choice of a specific IVAD, the usual consensus is to initiate therapy with midazolam or propofol, because they have a shorter duration of sedation and fewer side effects when compared with barbiturates. Individual patient characteristics, physician preference and experience, as well as local institutional practice also guide the choice of IVAD.

A recent systematic review 41 compared outcome measures associated with the initial choice of continuous IVAD in refractory SE. A total of 66 studies with 1,637 patients were included. Significant differences among IVADs – midazolam, propofol, and barbiturates – were observed in certain outcome measures. Short-term failure was more common with midazolam and propofol, while hypotension and dose-limiting hypotension were more frequent with barbiturates. Additionally, the need to switch IVAD was higher in patients initially treated with midazolam. In line with a previously published systematic review, 42 IVAD choice was not a predictor of mortality.

Midazolam is often the first choice to initiate the treatment of refractory SE due to its safety profile and widespread familiarity in most centers. However, a major drawback of midazolam in SE treatment is tachyphylaxis – the reduction in antiseizure effectiveness with prolonged use –, which can increase the likelihood of recurrent seizures and the need to switch the IVAD. 41 42 Withdrawal seizures and recurrence of SE are also relatively common, although this outcome may be improved with the use of higher doses. Fernandez et al. 43 compared two treatment protocols of refractory SE with continuous midazolam infusion: low-dose (median dose of 0.2 mg/kg/h) and high-dose (median dose of 0.4 mg/kg/h), both with similar duration of infusion. Withdrawal seizures (occurring within 48 hours of discontinuation of midazolam) were less frequent in the high-dose group; failure of midazolam treatment, requiring change to a different IVAD, as well as incidence of hospital complications, were not different between the groups. More importantly, patients treated with higher doses presented lower mortality.

Propofol is a gamma-aminobutyric acid (GABA) A receptor agonist with potent antiseizure effect. 44 The main concern regarding this drug is the propofol infusion syndrome (PRIS), consisting in rhabdomyolysis, severe metabolic acidosis, and cardiac and renal failures. 45 It is more common in children and in patients with acute neurological illnesses – especially traumatic brain injury 46 –or acute inflammatory diseases, complicated by severe infections or sepsis. It is associated with prolonged use (longer than 48 hours) and with infusion rates higher than 5 mg/kg per hour. 45 47 48 Special attention should be paid to patients receiving drugs with carbonic anhydrase inhibitor effect, such as acetazolamide and topiramate. Due to the risk of PRIS, the use of continuous propofol in children with refractory SE is limited. 48

Barbiturates are associated with a myriad of side effects, including hypotension, cardiac depression, hepatotoxicity, respiratory infections, ileus, and immune dysfunction. 49 When compared with midazolam and propofol, barbiturates more often cause hypotension leading to treatment discontinuation. 41 42 The half-lives of pentobarbital and thiopental may increase with prolonged use, mainly due to accumulation in adipose tissue, leading to prolonged sedation after weaning of the drug. In patients with recurrent seizures after the tapering of barbiturates, phenobarbital is a good option for maintenance therapy. 49

Data on the use of ketamine as the initial IVAD for refractory SE is scarcer. 41 Ketamine is an N-methyl-D-aspartate (NMDA) receptor antagonist, a mechanism of action that may be especially valuable in the later stages of SE, when GABAergic drugs – such as benzodiazepines, propofol, and barbiturates – have diminished effectiveness. 50 51 52 53 Recent data suggest that ketamine may be more effective than midazolam when administered as the first-line anesthetic infusion in pediatric SE, but this has yet to be confirmed by controlled studies. 54 55 Due to its mechanism of action and pharmacological characteristics, ketamine has even been suggested as a second-line option for the treatment of SE, after BZD failure, as an alternative to other ASMs. 56 Ketamine has a clinical advantage over the more commonly used IVADs: it rarely causes hypotension; therefore, it is useful in treating patients with hemodynamical instability. On the other hand, caution should be exercised when treating patients with hypertension. 50 Continuous IV infusion should be started with a loading dose of 2 mg/kg, followed by an infusion rate of 1 to 10 mg/kg/hour.

The infusion of continuous IVAD aims to control clinical as well as electrographic seizure. The appropriate titration of these drugs is only feasible with the aid of continuous EEG monitoring; if continuous monitoring is not available, daily EEG control is recommended. If EEG is not available at all, only recurrent clinical seizures will be diagnosed. After control of motor seizures in convulsive SE, electrographic seizures may occur in up to half of patients who do not recover normal consciousness. 57

There is no clear evidence to suggest any level of sedation or duration of therapeutic coma with continuous IVAD in refractory SE. Most guidelines suggest that coma should be induced for 24 to 48 hours. 11 12 58 However, this is based mainly on retrospectively collected data. Prolonged sedation increases the risk of complication and prolongs the length of stay in the ICU. 59 Also, coma induction with IVAD may be independently associated with prolonged hospitalizations, infection risk, and poor functional outcomes. 60 61 62 The use of IVADs in SE has been associated with increased in-hospital mortality, independently of confounding factors, 63 although this is still a matter of debate. 64 The EEG endpoint for continuous IVAD infusion is also not clearly defined. Many experts suggest that aiming at burst-suppression pattern on EEG is superior to the mere cessation of clinical and EEG seizures, but this strategy may carry a higher risk of complications. 42 65 Based on current evidence, it is reasonable to aim for the control of clinical and EEG seizures; if seizures recur after IVAD weaning, 24 to 48 hours of deeper sedation may be tried, aiming at burst-suppression on EEG.

Along with continuous infusion of IVAD, treatment with long-acting ASMs should be optimized. In addition to the ASM initially used, other ASMs may be added to achieve and maintain seizure control and to facilitate weaning off the continuous IVAD. Parenteral ASMs are preferred, as it is possible to achieve therapeutic serum levels more rapidly. Therefore, phenytoin and levetiracetam (if not previously administered), as well as valproate, lacosamide, and phenobarbital, can be loaded intravenously in this stage of the treatment. If needed, other ASMs, such as topiramate, clobazam, perampanel, and vigabatrin, can also be administered via a nasoenteric feeding tube.

The Treatments Committee of the American Epilepsy Society published a review 66 on the treatment of convulsive refractory SE, aiming to determine the strength of evidence for parenteral ASMs and other therapies used in special circumstances related to convulsive SE. The searches conducted by the committee identified no class-I, no class-II, 9 class-III, and multiple class-IV studies on the use of 13 ASMs in the treatment of convulsive refractory SE, highlighting the marked absence of high-quality data on this issue. The review 66 concluded that there is not enough data to support the efficacy of levetiracetam, valproate, lacosamide, brivaracetam, midazolam, propofol, pentobarbital, and ketamine either as the last ASM in treating convulsive refractory SE or compared to others of these drugs. Moreover, medications that have been used with variable efficacy in special situations, such as adrenocorticotropic hormone, intravenous immunoglobulin, corticosteroids, magnesium sulfate, and pyridoxine, have not been appropriately studied for convulsive refractory SE.

## Super-refractory status epilepticus

Super-refractory SE is defined as seizures persisting at least 24 hours after onset of anesthesia, either without interruption despite appropriate treatment with anesthesia, recurring while on appropriate anesthetic treatment, or recurring after withdrawal of anesthesia and requiring anesthetic reintroduction. 35 40 The IVADs used in the treatment of SE include midazolam, propofol, pentobarbital, thiopental, ketamine, and others, as long as they are used at anesthetic doses. Approximately 15% of all the cases of SE admitted to the hospital will become super-refractory. 40 If anesthetics are needed for at least 7 days, the term *prolonged super-refractory SE* applies, while the term *prolonged refractory SE* is used for SE of the same duration, but without the persisting need for anesthetics. 67 Super-refractory SE is a very severe condition, with high morbidity and mortality. The mortality rate increases the longer the episode of SE lasts, due to various complications of both the SE and its treatment: 40 68 with prolonged SE and treatment, complications such as hypotension, cardiorespiratory collapse and failure, hepatic failure, renal failure, disseminated intravascular coagulation and disorders of bleeding, infection, rhabdomyolysis, and ileus and gastrointestinal disturbances become progressively more incident.

When it comes to evidence-based medicine, the landscape for super-refractory SE is even more uncertain compared with refractory SE. Several points compromise the assessment of therapies which have been tried in super-refractory SE: first and foremost, the lack of randomized or controlled studies; the small number of individuals treated in the trials; co-medication and changing doses of co-medication; and publication bias. 69 In addition, super-refractory SE is a heterogeneous condition, in which factors such as age and etiology play a very important role, limiting the comparison of different treatments. 68 70

Ochoa et al. 71 reviewed studies on effectiveness of hypothermia, ketogenic diet (KD), vagus nerve stimulation (VNS), brain surgery, inhalational anesthetics, and other ASMs such as topiramate, pregabalin, lidocaine, and perampanel in the treatment of super-refractory SE. No class-I, 1 class-II, and multiple class-IV studies were identified on the use of therapeutic trials. After reviewing the available evidence, the authors 71 concluded that there is insufficient evidence that any of the ASMs reviewed, inhalational anesthetics, KD, acute VNS, brain surgery, or therapeutic hypothermia are effective treatments.

Therefore, when treating patients with super-refractory SE, one should bear in mind that it is likely that the patient has already received several different therapies and is already dealing with many clinical complications. At this point of treatment, given the lack of evidence to recommend a specific treatment, the choice of treatment should be individualized, and clinical judgement should be exercised in each case.

## NORSE and FIRES

Refractory and super-refractory SE may occur in patients with neither previous history of epilepsy nor clearly-defined etiology. New-onset refractory SE (NORSE) is a clinical presentation, not a specific diagnosis, in a patient without active epilepsy or other preexisting relevant neurological disorders, with new onset of refractory SE without a clear acute or active structural, toxic, or metabolic cause. 35 More often, NORSE is super-refractory. Febrile infection-related epilepsy syndrome (FIRES) is a subcategory of NORSE that requires a previous febrile infection, with fever starting between 2 weeks and 24 hours prior to the onset of refractory SE, with or without fever at the onset of the SE. 35 Both definitions apply to all age groups. If no explanation for the clinical presentation of NORSE (or FIRES) is found after diagnostic workup, cryptogenic NORSE is defined.

Both NORSE and FIRES present a significant treatment challenge, particularly because, in the absence of a known etiology, the options to target the underlying cause of the seizures are limited. There are no randomized controlled trials or consensus guidelines for the diagnostic evaluation and treatment of patients who present with NORSE/FIRES. A group of international experts in this field proposed a series of recommendations 72 for the diagnostic evaluation and management of NORSE/FIRES. An extensive diagnostic evaluation is recommended, aiming at defining the underlying cause of SE. In cases of cryptogenic NORSE, if SE persists after 48 to 72 hours, immunotherapy with methylprednisolone or intravenous immunoglobulin (IVIg) should be considered. In cases of incomplete response, second-line immunotherapy and/or KD should be considered after 7 days. The choice of the therapy depends on the clinical presentation. 72 73

In conclusion, SE is a relatively common and severe condition associated with high morbidity and mortality. Prompt recognition is essential to ensure the timely initiation of the appropriate treatment. From the outset, patient stabilization and correct administration of ASMs must be prioritized. Additionally, identifying the underlying cause is crucial, and a thorough diagnostic workup should be conducted alongside treatment.

In treating patients with SE, the well-known aphorism from vascular neurology applies: “time is brain.” Delays in recognizing and treating SE are associated with poorer outcomes. During the initial stage of therapy, timely administration of BZDs at correct doses is essential. Following BZD use, IV ASM should be administered. In Brazil, IV levetiracetam is now available as an option, alongside phenytoin. If convulsive seizures persist after BZDs and IV ASM, the next step is to induce therapeutic coma using IVADs.

Treating refractory and super-refractory SE is challenging, as evidence supporting specific treatments remains limited. In the absence of randomized controlled trials, treatment should rely on the physician's experience, clinical judgment, and previously reported therapeutic options. Some patients may achieve good outcomes despite prolonged SE; therefore, intensive treatment should be continued as long as a favorable outcome remains possible.
